# Editorial: Minimally invasive surgery in benign gynecological pathology

**DOI:** 10.3389/fmed.2024.1379505

**Published:** 2024-02-22

**Authors:** Laurentiu Pirtea

**Affiliations:** Department of Obstetrics and Gynaecology, Victor Babes University of Medicine and Pharmacy, Timisoara, Romania

**Keywords:** laparoscopy, gynecology, robotic surgery, hysteroscopy, minimally invasive surgery

In the ever-evolving landscape of medical science, one particularly remarkable advancement has changed the surgical approach of the abdominal wall—minimally invasive surgery.

This revolutionary approach has transformed the field of gynecology by offering safer, less painful and more effective alternatives to traditional open surgery. In this editorial, we will explore the significance of minimally invasive surgery in treating benign gynecological conditions and the positive impact it has on patients' quality of life after complex surgery.

Traditionally, benign gynecological pathologies such as uterine fibroids, ovarian cysts, endometriosis and pelvic adhesions often required open abdominal surgeries, which involved large incisions, extended hospital stay and prolonged recovery periods. These procedures also implied a higher risk of complications, increased pain and emotional distress for patients.

The philosophy of minimally invasive surgery is performing complex surgery for various pathologies through small incisions. This can be achieved by laparoscopy, robotic and vaginal surgery. In the spirit of reducing the surgical related trauma the use of natural orifices such as the vagina to approach the pelvis is very effective. Xie et al. showed that surgery for ovarian cyst can be performed by transvaginal natural orifice endoscopic surgery as a day-care procedure. Mei et al. performed the comparison of gases and traditional robot-assisted transvaginal natural orifice transluminal endoscopic surgery in hysterectomy showing that there is still room for reducing the trauma associated to minimal invasive surgery.

One of the most common benign gynecological conditions treated using minimally invasive surgery is uterine fibroids. This represents the most common benign disease of the uterus, affecting up to 68.6% women (1) generating symptoms like heavy menstrual bleeding, pelvic pain, and infertility. Minimal invasive surgery can be used to perform either hysterectomy or myomectomy (1–6). Dumitraşcu et al. performed a review showing the importance of surgical technique when performing laparoscopic myomectomy. This approach is suitable for complex cases also, and techniques to minimize blood loss during surgery can be applied (7–13) Balulescu et al. presented the results of a clinical trial that investigated the efficiency of temporary occlusion of the hypogastric artery during laparoscopic myomectomy (9). Hertling et al. demonstrated the benefit of uterine artery embolisation before laparoscopic myomenucleation of large fibroids.

Pelvic floor disorders that require complex surgery can also be approached by minimal invasive surgery. Ciortea et al. performed a systematic review and meta-analysis investigating the best approach for the treatment of vaginal vault prolapse and showing the benefits of minimal invasive surgery.

Hysteroscopy is another form a minimal invasive surgery (14–18) and the spectrum of indication for this type of surgery is developing continuously. Teng et al. published their results with hysteroscopic curettage in the treatment of type II cesarean scar pregnancy. Liu et al. investigated the use of hysteroscopy vs. dilation and curettage for the assessment of the endometrium.

The advantages of minimally invasive surgery extend beyond the operating room. Patients experience shorter hospital stays, reduced postoperative pain, faster recovery, and improved cosmetic outcomes due to smaller incisions. Furthermore, the risk of postoperative complications, such as infection and blood loss, is significantly lower compared to traditional open surgeries (19–23). Xing et al. demonstrated that enhanced recovery after surgery alleviates neutrophil to lymphocyte ratio, correlates with lower pain score and faster recovery.

Hence, minimally invasive surgery leads to better overall patient satisfaction. Women who undergo these procedures report improved quality of life, as they can return to their daily activities sooner and with less discomfort. This is particularly crucial for those who are balancing their careers, families, and personal lives.

While *Minimally invasive surgery in benign gynecological pathology* has transformed the field, it is essential to acknowledge that these techniques require specialized training and experience. Surgeons must be skilled in the use of laparoscopic and robotic-assisted equipment to ensure optimal outcomes for patients. Therefore, continued investment in surgical education and technology is paramount to further advancing the field.

In conclusion, *Minimally invasive surgery in benign gynecological pathology* has ushered in a new era of patient-centered care. It has replaced the traditional open surgeries with less invasive, safer, and more effective options. Women facing conditions such as uterine fibroids, ovarian cysts, endometriosis, and pelvic adhesions can now benefit from quicker recoveries, shorter hospital stays, and improved overall wellbeing. As we continue to advance in the field of gynecology, the integration of minimally invasive techniques will undoubtedly play a pivotal role in empowering women to lead healthier, more fulfilling lives.

## Author contributions

LP: Writing—original draft, Writing—review & editing.
